# Construction of Uniform Thermal Network in Passive Radiative Cooling Coatings by Nanocellulose-Mediated Pickering Emulsion Template

**DOI:** 10.3390/polym18202591

**Published:** 2026-10-21

**Authors:** Huiwen Pang, Jiaqi Zhu, Zenghui Xu, Tong Wang, Xiang Li, Xing Chen, Niancheng Hong

**Affiliations:** 1 China Construction Eco-Development New Energy Co., Ltd., Nanjing 210012, China; 2 Center for Composite Materials and Structures, School of Astronautics, Harbin Institute of Technology, Harbin 150006, China; 3 Research & Development Center, China Construction Eco-Development Group Co., Ltd., Suzhou 215000, China

**Keywords:** radiative cooling coating, Pickering emulsions, TEMPO-oxidized cellulose nanofibrils, thermal conductivity, hexagonal boron nitride

## Abstract

Developing high-performance passive radiative cooling coatings (PRCC) with high thermal conductivity for fast heat dissipation from self-heating objects remains a significant challenge. In this study, a nanocellulose-mediated Pickering emulsion strategy was employed to construct a uniform hexagonal boron nitride (BN) thermal network within PRCC. Benefiting from the Pickering emulsion template stabilized by TEMPO-oxidized cellulose nanofibrils (TOCNF), BN nanosheets and other functional inorganic nanoparticles were uniformly dispersed in the PRCC matrix, forming a continuous and homogeneous thermally conductive network. This structural design endowed the PRCC with excellent thermal conductivity, high solar reflectance in the solar spectrum (0.3–2.5 μm), and strong emissivity in the “atmospheric window” (ATSW, 8–13 μm). Specifically, the thermal conductivity of the TOCNF-BN-modified PRCC reached 2.28 ± 0.15 W·m^−1^·K^−1^, which was significantly higher than that of the pristine PRCC. The coating exhibited a solar reflectivity of ~87% and an emissivity of ~93% in the ATSW. Outdoor model temperature tests indicated that the internal temperature of the PRCC-TOCNF-BN-coated metal cabinet was 9.6 °C lower than that of the bare cabinet and 6.7 °C lower than the cabinet coated with pristine PRCC. This novel approach of using Pickering emulsion templates to fabricate highly thermally conductive radiative cooling coatings provides a new strategy for the development of high-performance and multifunctional radiative cooling materials for thermal management applications.

## 1. Introduction

The escalating global energy crisis and accelerating environmental degradation have rendered sustainable, low-carbon development imperative [1,2]. Conventional active cooling technologies, which account for approximately 15% of global electricity consumption and contribute substantially to greenhouse gas emissions, are increasingly unsustainable [3,4]. Radiative cooling technology has emerged as a promising passive thermal management strategy that requires zero energy input and produces no environmental pollutants [5,6]. This process leverages the atmospheric transmission window (ATSW, 8–13 μm) to discharge thermal radiation directly toward outer space (~3 K), which enables sub-ambient cooling without external power supply [7,8].

Recent advances in radiative cooling materials—including photonic, polymeric, and coating formats—have pushed solar reflectance and atmospheric-window emissivity to practical levels [9]. Among these radiative cooling material formats, polymer-based coatings stand out for their solution processability, low cost, and adaptability to surfaces of arbitrary geometry [10,11,12,13]. However, conventional passive radiative cooling coatings (PRCC) are primarily designed for thermally passive objects operating near ambient temperatures, and their performance degrades significantly when applied to self-heating infrastructure such as telecommunication base stations, data centers, and concentrated photovoltaic systems [14,15]. This limitation stems from a fundamental materials paradox: (i) the inherently low thermal conductivity of polymer matrices, which is further exacerbated by the increased coating thickness required for sufficient solar reflectance, and (ii) the resulting thermal resistance that causes heat accumulation, effectively nullifying the radiative cooling effect. Addressing this bottleneck necessitates the simultaneous optimization of solar reflectance, thermal emittance, and thermal conductivity (both through-plane transport and in-plane spreading) of the coating—a tripartite challenge that remains largely unresolved [16,17].

The incorporation of high-thermal-conductivity nanofillers, such as hexagonal boron nitride (BN), aluminum nitride, and graphene, represents an effective strategy for enhancing thermal transport in polymer composites [18,19]. However, the random dispersion of fillers typically results in agglomeration and the formation of discontinuous heat flow networks [20]. Within such networks, phonons undergo severe scattering at anisotropic heat-flow pathways, non-uniform crystalline domains, and polymer–filler interfaces, leading to interfacial thermal resistances that markedly degrade the effective thermal conductivity [21]. Theoretical models suggest that constructing a continuous, three-dimensionally interconnected thermally conductive network is essential to minimize phonon scattering and achieve efficient heat dissipation at low filler loadings [22,23].

Interfacial assembly strategies, particularly Pickering emulsion templating, have recently demonstrated exceptional efficacy in establishing such networks within polymer matrices [24,25]. Pickering emulsions, stabilized by solid particles rather than molecular surfactants, enable the precise spatial organization of functional fillers at the oil–water interface [26]. However, inorganic nanofillers employed in radiative cooling coatings, including BN, TiO_2_, and SiO_2_, cannot independently stabilize at the liquid–liquid interface due to their intrinsic wettability characteristics [27]. Recent advances have demonstrated that TEMPO-oxidized cellulose nanofibrils (TOCNF)—characterized by their high aspect ratio, uniform nanoscale dimensions, and high surface charge density—can serve as effective emulsifiers and facilitate the uniform dispersion of inorganic nanofillers [28,29]. The amphiphilic nature of TOCNF, arising from hydrophobic cellulose backbones and hydrophilic surface-functionalized groups, enables their adsorption at the water–oil interface, thereby stabilizing nanofillers and preventing agglomeration during film formation [30].

In this work, we present a nanocellulose-mediated Pickering emulsion strategy for the construction of uniform and continuous BN thermal networks within passive radiative cooling coatings. Using TOCNF as both an emulsion stabilizer and a structural template, we achieve the homogeneous dispersion of BN nanosheets in a waterborne acrylic matrix incorporated with titanium dioxide and silicon dioxide as optical functional fillers. The structural evolution and formation mechanism of the thermal network are systematically investigated, and the role of the Pickering templating effect in regulating the dispersion state and interfacial thermal resistance of the thermally conductive fillers is elucidated. This study provides a versatile and scalable approach to decouple thermal transport and optical performances in radiative cooling materials, offering a promising pathway for advancing their applications in high-power thermal management systems.

## 2. Materials and Methods

### 2.1. Materials

Waterborne acrylic emulsion (50 wt%), SiO_2_ (99.9%), TiO_2_ (99.9%), ammonium salt of poly(acrylic acid)-b-styrene block copolymer (99.9%), hydroxyethyl cellulose (5000–6400 mPa·s), polyether-modified polydimethylsiloxane (99.9%), 2,2,4-trimethyl-1,3-pentanediol mono-isobutyrate (99.9%), dimethylethanolamine (99.5%), and octylphenol ethoxylate-10 (99.9%) were sourced from Weihai Kaimitek Trading Co., Ltd (Weihai, China). Hexagonal boron nitride (BN, 99.9%) was procured from Hebei Wenlun Metal Materials Co., Ltd. (Cangzhou, China). TEMPO-oxidized cellulose nanofibrils (TOCNF, carboxylate content 1.2 mmol/g), quaternary ammonium salt-modified cellulose nanofibrils (NCNF, quaternary ammonium content 0.8 mmol/g), sulfonated cellulose nanofibrils (SCNF, sulfonate content 1.0 mmol/g), and phosphorylated cellulose nanofibrils (PCNF, phosphate content 0.9 mmol/g) were supplied by Tianjin Wood Spirit Biotechnology Co., Ltd. (Tianjin, China). All reagents were used as received without further purification; deionized water served as the solvent throughout.

### 2.2. Preparation of Modified Radiative Cooling Coatings

#### 2.2.1. Preparation of Pickering Emulsion

The Pickering emulsion was produced by mechanical shear emulsification. Briefly, 10.0 g of waterborne acrylic emulsion was combined with 5.0 g of deionized water under magnetic stirring at 500 rpm for 10 min. TOCNF (0.5 wt% relative to total solids) was then added and dispersed by ultrasonication at 150 W/30 kHz for 20 min. BN (5 wt%), TiO_2_ (20 wt%), and SiO_2_ (10 wt%), all relative to total solid content, were sequentially introduced. High-shear emulsification at 10000 rpm for 30 min on an IKA T25 homogenizer yielded a stable Pickering emulsion. Post-emulsification, 0.3 g of hydroxyethyl cellulose (thickener), 0.1 g of polyether-modified polydimethylsiloxane (defoamer), 0.2 g of dimethylethanolamine (pH adjuster), and appropriate amounts of poly(acrylic acid)-b-styrene ammonium salt (dispersant), 2,2,4-trimethyl-1,3-pentanediol monoisobutyrate (coalescent), and octylphenol ethoxylate-10 (surfactant) were blended in, followed by stirring at 800 rpm for 15 min to obtain a homogeneous slurry. Control samples modified with NCNF, SCNF, or PCNF, as well as specimens lacking cellulose nanofibrils or BN, were prepared identically [31].

#### 2.2.2. Fabrication and Basic Performances of the Coatings

The slurry was cast onto cleaned substrates (100 mm × 100 mm × 2 mm) with a doctor blade set to a 200 μm wet film thickness. After drying at 60 °C for 2 h to remove water and solvents, the films were cured at 120 °C for 1 h, yielding dense coatings with a controlled dry thickness of 80–100 μm. Slurry viscosity was recorded on a Brookfield DV2T rotational viscometer (Middleboro, MA, USA) at 25 °C. Slurry pH was measured with a Mettler Toledo FE28 m (Greifensee, Zürich, Switzerland) at 25 °C.

### 2.3. Structural Characterizations

Surface and cross-sectional morphologies were captured by scanning electron microscopy (SEM; Hitachi SU8010, Hitachi High-Technologies Corp., Tokyo, Japan) at 5 kV. Energy-dispersive X-ray spectroscopy (EDX) elemental mapping documented the spatial distribution of constituent elements. Fourier-transform infrared (FT-IR) spectra were acquired on a Thermo Scientific Nicolet 6700 spectrometer (Thermo Fisher Scientific Inc., Waltham, MA, USA) via the attenuated total reflection (ATR) mode (600–4000 cm^−1^, 4 cm^−1^ resolution, 32 scans). X-ray diffraction (XRD) patterns were collected on a Bruker D8 ADVANCE diffractometer (Bruker AXS GmbH, Karlsruhe, Germany) with Cu Kα radiation (λ = 0.154 nm). X-ray photoelectron spectroscopy (XPS) was performed on a Thermo Escalab 250 spectrometer (Thermo Fisher Scientific Inc., East Grinstead, UK) using Al Kα radiation (1486.6 eV); high-resolution spectra were deconvoluted with XPS Peak 4.1 software applying Shirley background subtraction.

### 2.4. Optical Performance Test

Solar reflectance (0.3–2.5 μm) was determined on a PerkinElmer Lambda 950 UV-Vis-NIR spectrophotometer (PerkinElmer Inc., Waltham, MA, USA) equipped with an integrating sphere; BaSO_4_ served as the reference. ATSW emissivity (8–13 μm) was measured at 25 °C on a Thermo Scientific Nicolet iS50 FT-IR spectrometer (Thermo Fisher Scientific Inc., Waltham, MA, USA) fitted with an emissivity accessory.

### 2.5. Thermal Conductivity Test

Thermal conductivity (λ) was derived from λ = α × ρ × cp, where α, ρ, and cp denote thermal diffusivity, density, and specific heat capacity, respectively. The through-plane thermal diffusivity was measured on a Netzsch LFA 467 laser flash analyzer (NETZSCH-Gerätebau GmbH, Selb, Germany) at 25 °C following ASTM E1461 [32] (specimen size 10 mm × 10 mm × 0.1 mm; three independently prepared specimens per formulation, five laser shots per specimen; both faces graphite-coated; instrument verified against Pyroceram 9606/NIST SRM 1461 within ±2.0%). Density was obtained by the Archimedes method (ASTM D792; weighing in air and in degassed deionized water at 25 °C). Specific heat was determined on a Netzsch DSC 214 differential scanning calorimeter (NETZSCH-Gerätebau GmbH, Selb, Germany) at 25 °C (heating rate 10 °C/min) with sapphire as reference per ASTM E1269. Reported values are mean ± standard deviation over the three independent specimens; the raw data are compiled in Supplementary Table S1.

#### Outdoor Temperature Reduction Test

Outdoor field measurements were carried out in Suzhou, Jiangsu Province, China (31.3° N, 120.6° E) on 15 May 2026 under clear-sky conditions. The peak solar irradiance reached above 850 W/m^2^, and the maximum ambient temperature reached 27.7 °C. Three geometrically identical metal cabinets (300 mm × 250 mm × 140 mm, wall thickness 1.0 mm) served as test models. Cabinet 1 received PRCC-TOCNF-BN on all exterior surfaces (dry thickness 80–100 μm). Cabinet 2 was coated with pristine PRCC at the same thickness. Cabinet 3 remained bare as the baseline. All three were placed on elevated polyurethane foam platforms (λ < 0.03 W·m^−1^·K^−1^, thickness 100 mm) to suppress ground conduction, oriented with their largest face (300 mm × 250 mm) perpendicular to direct solar radiation. Each internal cavity was kept empty so that solar-driven heating constituted the sole thermal load.

K-type thermocouples (±0.5 °C accuracy, <5 s response) were arranged as follows: two were bonded at the geometric center of each cabinet’s outer top surface; two more were suspended at the geometric center of the internal air cavity via PTFE brackets to avoid wall conduction artifacts. Ambient temperature was recorded by a shielded platinum resistance thermometer (±0.2 °C accuracy) positioned 1.5 m above ground.

## 3. Results and Discussion

### 3.1. Design Strategy for Radiative Cooling Coatings

**Scheme 1:**
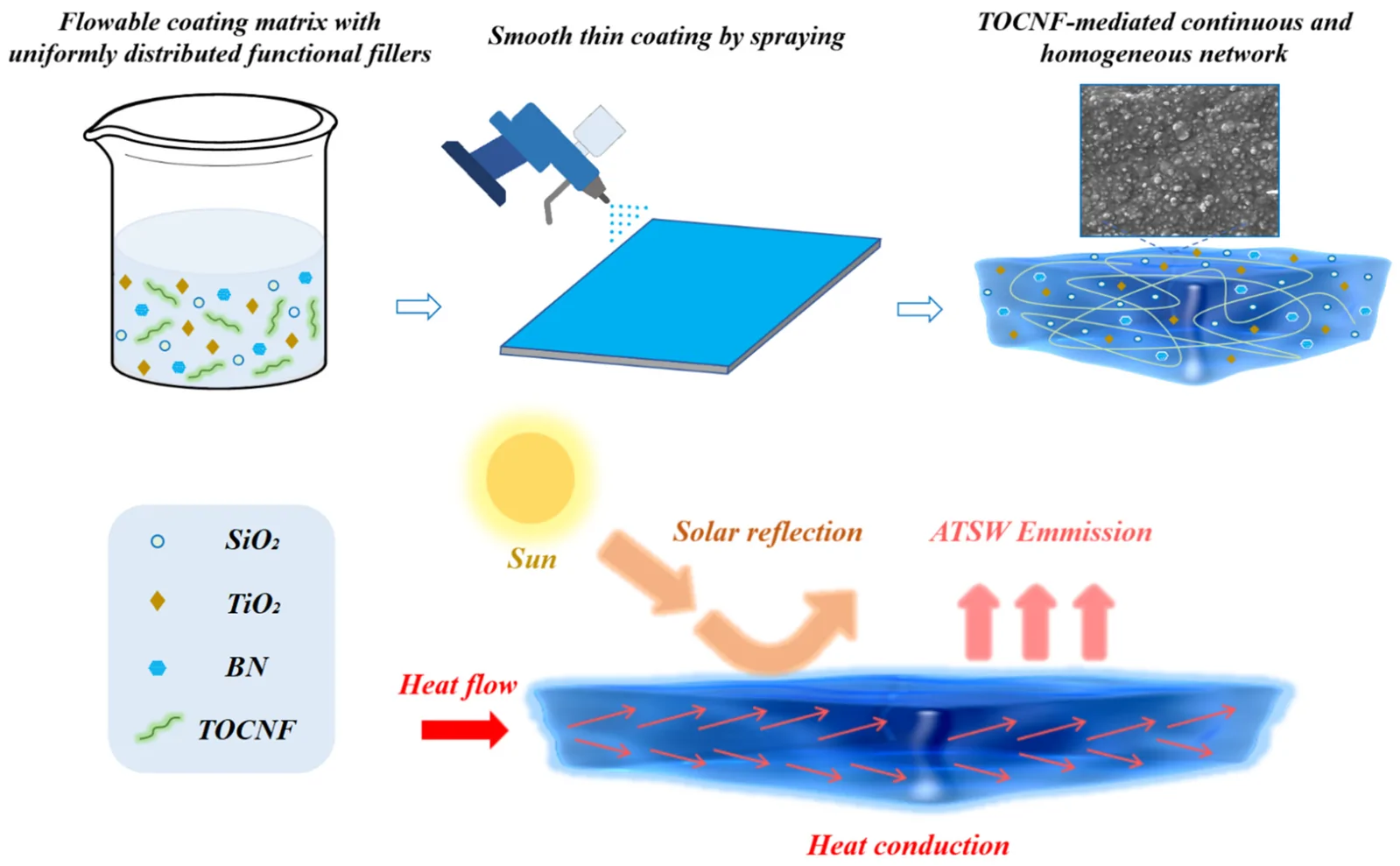
Schematic illustration of the design strategy for the PRCC-TOCNF-BN coating.

The PRCC-TOCNF-BN design concept is outlined in Scheme 1. TOCNF acts as a Pickering emulsion structural template that stabilizes the water–oil boundary of the waterborne acrylic emulsion. Under mechanical shear, TOCNF adsorbs at the interface, arresting droplet coalescence and securing uniform dispersion of the functional fillers (BN, TiO_2_, SiO_2_) within the matrix; a smooth, thin coating is obtained after spraying. TiO_2_ serves as the solar reflective filler—its high refractive index enables strong Mie scattering across the solar spectrum. SiO_2_ and BN function as infrared emitters, each contributing to high ATSW emissivity via phonon-polariton resonance and intrinsic phonon modes, respectively. Beyond stabilizing the emulsion, TOCNF also builds a hierarchical micro-nano surface texture upon curing, augmenting light scattering and solar reflectance. Furthermore, TOCNF provides a structural scaffold that directs BN nanosheets into a continuous thermal network, streamlining phonon transport and raising thermal conductivity. The combined action of filler intrinsic properties, their micro/nano architectures, and the TOCNF-mediated continuous thermal pathway endow the coating with both superior radiative cooling capability and enhanced thermal conductivity.

### 3.2. Impact of CNF on the Basic Performances of the Coatings

**Figure 1:**
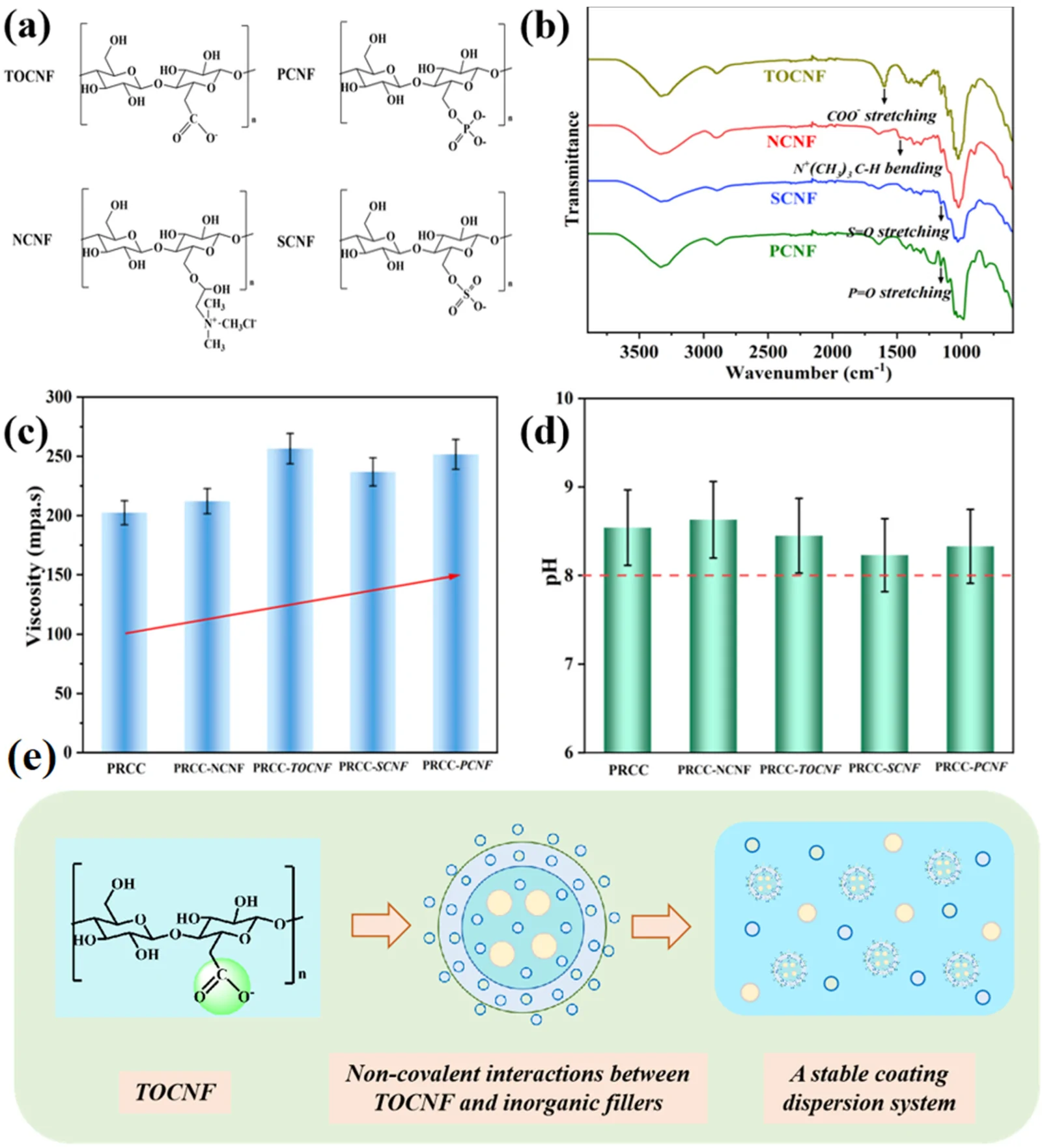
Impact of CNFs on the basic performances of the coatings: (**a**) molecular structures of four types of CNFs; (**b**) FT-IR spectra of four types of CNFs; (**c**) Viscosity of the CNF-modified PRCCs; (**d**) pH values of the CNF-modified PRCCs; (**e**) mechanism of TOCNF-assisted dispersion of inorganic fillers.

Four CNF variants (TOCNF, NCNF, SCNF, PCNF) were evaluated as PRCC modifiers, and their influence on fundamental coating properties is summarized in Figure 1. Their molecular architectures (Figure 1a) share a cellulose backbone but differ in functional side chains: carboxylate (−COO^−^) for TOCNF, quaternary ammonium (−N^+^(CH_3_)_3_) for NCNF, sulfonate (−SO^3−^) for SCNF, and phosphate (−PO_3_^2−^) for PCNF. FT-IR spectra (Figure 1b) corroborated the characteristic absorptions of each variant: 1595 cm^−1^ (COO^-^ stretching, TOCNF), 1478 cm^−1^ (N^+^(CH_3_)_3_ C–H bending, NCNF), 1182 cm^−1^ (S=O stretching, SCNF), and 1085 cm^−1^ (P=O stretching, PCNF) [33].

Viscosity measurements (Figure 1c) revealed that CNF addition markedly raised slurry viscosity. TOCNF produced the most pronounced effect: 256.48 mPa·s, a 2.3-fold increase over the unmodified PRCC (111.25 mPa·s). This enhancement arises from non-covalent interactions—chiefly hydrogen bonding—between CNFs and the polymer matrix, which amplify internal friction. All slurries displayed shear-thinning behavior: agitation with a glass rod transiently lowered viscosity by disrupting the internal network and associated non-covalent bonds [34]. The pH profiles of CNF-modified PRCC are presented in Figure 1d. The unmodified PRCC registered a pH of 8.54. NCNF addition raised the value to 8.63 owing to the alkaline quaternary ammonium functionality. Conversely, TOCNF, SCNF, and PCNF each slightly lowered pH to 8.32, 8.28, and 8.35, respectively, consistent with their acidic surface groups. All values fell within the 8.0–8.7 window deemed suitable for coating application. Taking into account both viscosity enhancement and component compatibility, TOCNF was selected as the preferred Pickering emulsifier for the remainder of this study.

### 3.3. Internal Structure of the Coatings

**Figure 2:**
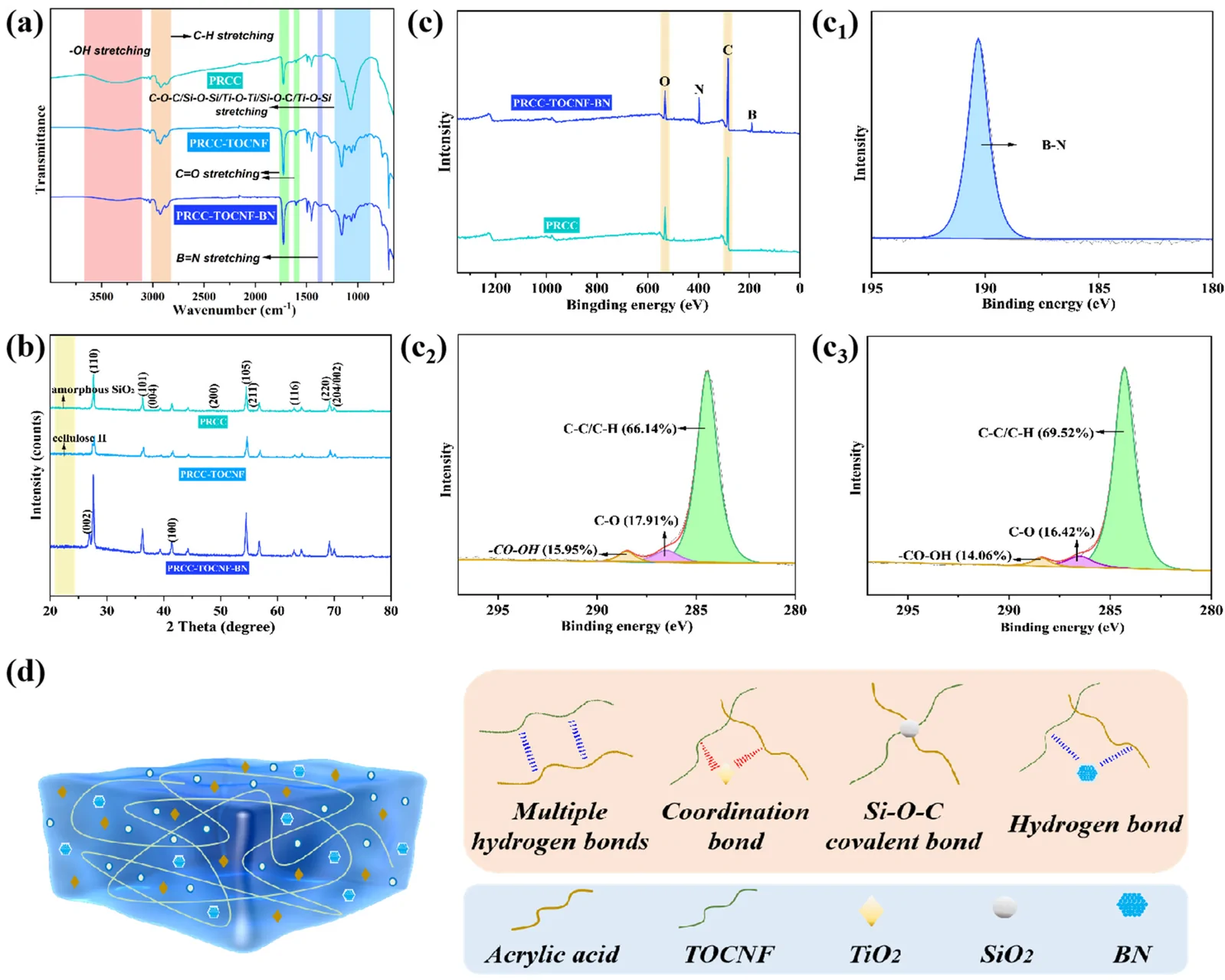
Intermolecular interactions and structural characteristics of the coatings: (**a**) FT-IR spectra; (**b**) XRD patterns; (**c**) XPS full-scan spectra, high-resolution B 1s spectra of PRCC-TOCNF-BN (**c1**), and high-resolution C 1s spectra of PRCC (**c2**) and PRCC-TOCNF-BN (**c3**). (**d**) Schematic illustration of the interfacial interactions and bonding modes among acrylic acid, TOCNF, TiO_2_, SiO_2_, and BN.

FT-IR spectra of PRCC, PRCC-TOCNF, and PRCC-TOCNF-BN are compiled in Figure 2a. The unmodified PRCC displayed characteristic absorptions at 3112–3671 cm^−1^ (−OH stretching), 2828–3010 cm^−1^ (C–H stretching), 1724 cm^−1^ (C=O stretching), and 876–1220 cm^−1^ (C–O–C/Si–O–Si/Ti–O–Ti/Si–O–C/Ti–O–Si stretching). Upon TOCNF incorporation, the C=O stretching intensity rose owing to the introduced carboxylate groups, whereas the −OH band weakened, signaling hydrogen-bond formation between TOCNF and the polymer matrix. With BN present (PRCC-TOCNF-BN), a new peak at 1368 cm^−1^ corresponding to B=N stretching confirmed BN incorporation; the further attenuation of the −OH band pointed to strong hydrogen-bonding between TOCNF and BN surfaces [35].

XRD patterns are presented in Figure 2b. Pristine PRCC showed sharp reflections at 2θ = 27.4°, 36.1°, 37.8°, 48.0°, 53.9°, 55.1°, 62.7°, 68.8°, and 70.3°, corresponding to the (110) and (101) planes of rutile TiO_2_, the (004), (200), (105), (211), (116), and (220) planes of anatase TiO_2_, and the overlapping anatase (204)/rutile (002) reflections, respectively, indicating the coexistence of anatase and rutile phases. A broad hump centered at 2θ ≈ 22 ° indicated amorphous SiO_2_. The crystallinity index (CI) of PRCC stood at 0.68. PRCC-TOCNF introduced a weak shoulder at 2θ ≈ 22.6 ° from cellulose II, partially overlapping a TiO_2_ peak; CI dropped to 0.52, ascribed to the amorphous TOCNF component [36]. PRCC-TOCNF-BN added new peaks at 2θ = 26.8 ° and 41.6 °, indexed to the (002) and (100) planes of BN [37]. CI fell further to 0.45, reflecting the dilution and peak-overlap effects of the added crystalline BN and semi-crystalline TOCNF on the integrated TiO_2_ reflections, together with the suppression of TiO_2_ grain growth during curing. The pronounced relative intensity of the BN (002) reflection indicates a partial (00l) preferred orientation of the platelets—basal planes preferentially parallel to the film plane—induced by shear emulsification and film formation, while the retained (100) intensity confirms that a fraction of tilted and edge-on platelets bridge the aligned ones through edge-to-face contacts, yielding the quasi-3D interconnected network.

XPS analysis of PRCC and PRCC-TOCNF-BN is displayed in Figure 2c. Survey spectra confirmed that PRCC-TOCNF-BN contains C, O, Ti, Si, B, and N, whereas PRCC contains only C, O, Ti, and Si, verifying BN incorporation. The high-resolution B 1s spectrum of PRCC-TOCNF-BN featured a lone peak at 190.2 eV corresponding to B–N bonding, indicating that BN retained its structural integrity without oxidation. Deconvolution of the PRCC C 1s spectrum yielded three components: C–C/C–H (284.8 eV, 66.14%), C–O (286.4 eV, 17.91%), and O–C=O/−COOH (288.6 eV, 15.95%) [29]. In PRCC-TOCNF-BN, the C–C/C–H fraction rose to 69.52%, while C–O and O–C=O/−COOH fell to 16.42% and 14.06%, respectively—a shift accounted for by TOCNF introduction expanding the non-oxygenated carbon domain proportion [35,38,39].

Figure 2d schematically summarizes the key interfacial interactions—multiple hydrogen bonds, coordination bonds, Si–O–C covalent linkages, and hydrogen bonding—among acrylic acid, TOCNF, TiO_2_, SiO_2_, and BN, which stabilize the Pickering emulsion and guide the formation of the continuous thermally conductive network.

**Figure 3:**
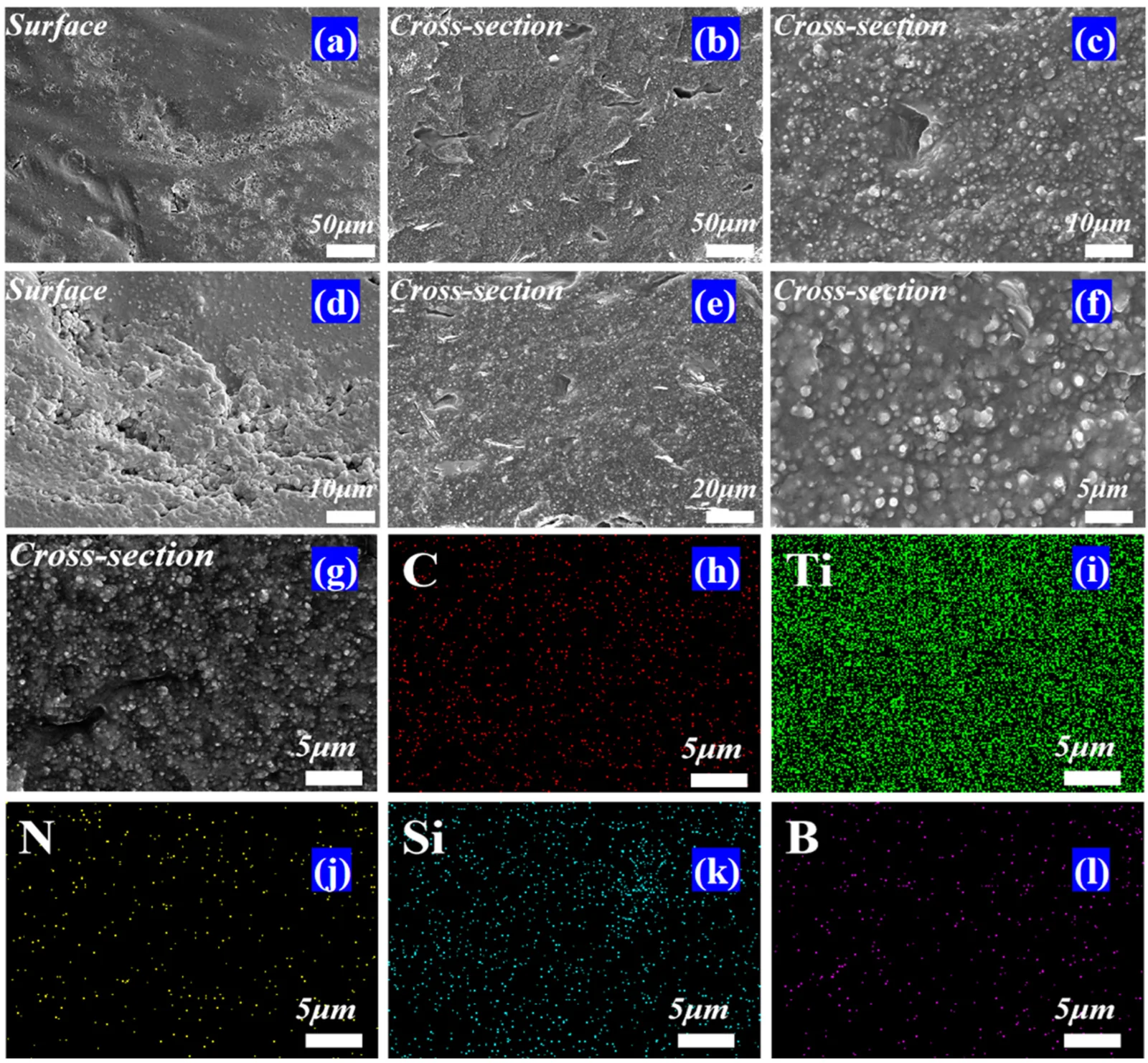
SEM images and EDX elemental mapping of PRCC-TOCNF-BN: (**a**–**f**) surface and cross-section morphologies at different magnifications; (**g**–**l**) EDX elemental mapping of C, Ti, N, Si, and B elements.

Surface and cross-sectional SEM images of PRCC-TOCNF-BN are compiled in Figure 3a–f. The coating surface appeared fairly compact, with a few microscopic voids likely stemming from air entrapment during spraying [40]. Cross-sectional views revealed a dense, pore-free interior, confirming robust cross-linking. Micro-scale protrusions on the fracture surface corresponded to embedded TiO_2_ and SiO_2_ nanoparticles, and fibrous TOCNF strands were visible throughout, serving as a structural scaffold. EDX elemental mapping (Figure 3g–l) demonstrated uniform distribution of C, Ti, Si, B, and N, corroborating the homogeneous dispersion of BN, TiO_2_, and SiO_2_. Localized enrichment of B and N pointed to BN microcrystalline domains that underpin the continuous thermal conductive network [19,41]. At the highest magnification, individual BN nanosheets and their edge-to-face contacts are clearly resolved, directly visualizing the interconnected platelet network. The B and N EDX signals extend homogeneously across the entire coating thickness, confirming the three-dimensional continuity of the BN network rather than its confinement to discrete layers.

### 3.4. Optical Performances of the Coatings

**Figure 4:**
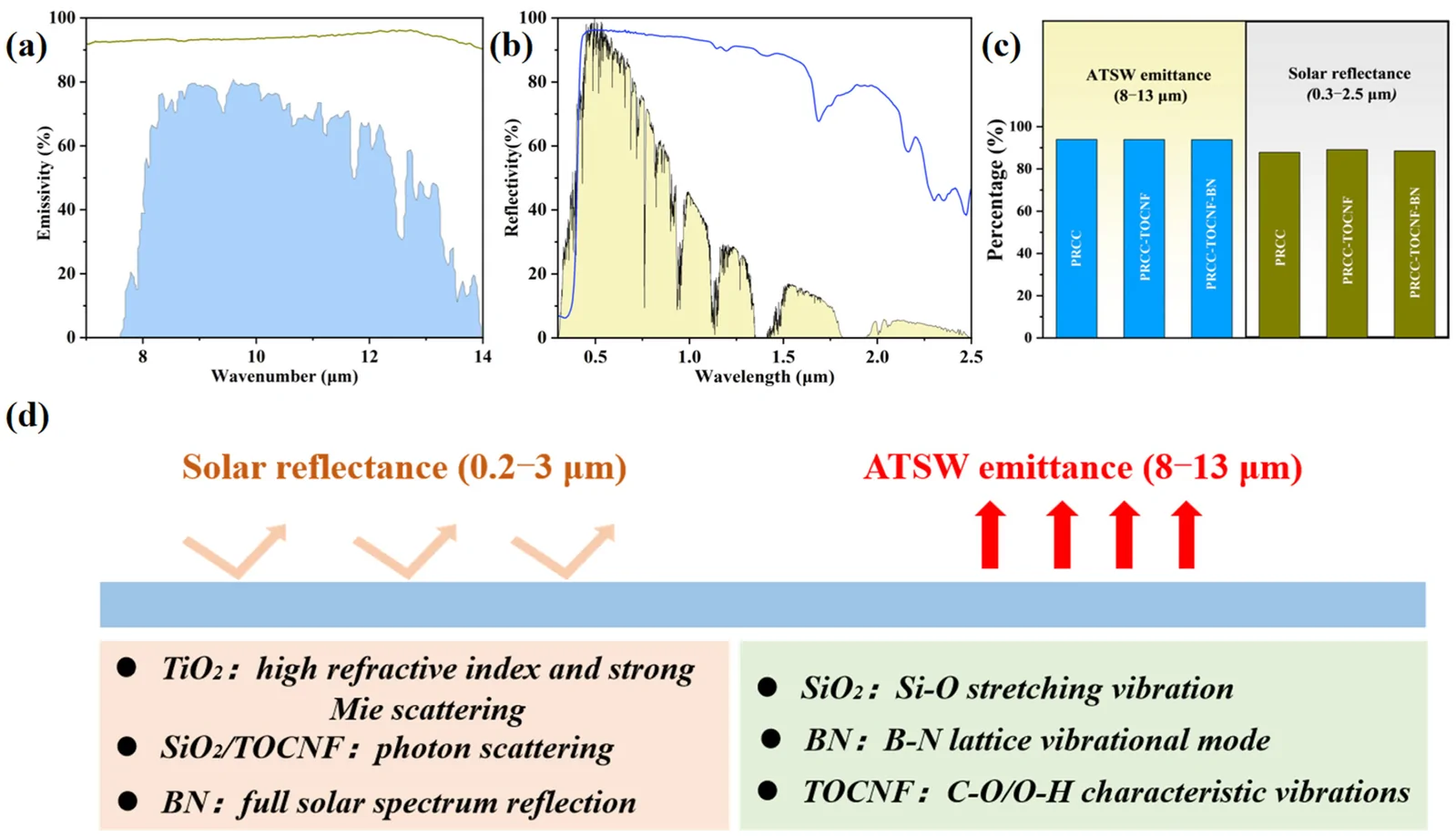
Optical performances of the coatings: (**a**) emissivity spectrum in the 8–13 μm range; (**b**) solar reflectance spectrum in the 0.3–2.5 μm range; (**c**) comparison of solar reflectance and ATSW emissivity; (**d**) schematic illustration of the optical mechanism.

Optical performance data are presented in Figure 4. PRCC-TOCNF-BN achieved a solar reflectance of approximately 87% across 0.3–2.5 μm (Figure 4b) and an ATSW emissivity of approximately 93% (Figure 4a). The high solar reflectance derives principally from Mie scattering by TiO_2_ nanoparticles—their refractive index (*n* ≈ 2.5) and size distribution enable efficient scattering across the solar spectrum. The hierarchical micro-nano architecture created by TOCNF amplifies reflectance through synergistic photon scattering with TiO_2_/SiO_2_, sustaining reflectance above 85% even in the near-infrared. BN contributes broadband solar reflection that further bolsters overall reflectance [13,42].

The elevated ATSW emissivity arises from several synergistic mechanisms: SiO_2_ phonon-polariton resonance (Si–O stretching vibration) generates strong dipole oscillations in the mid-infrared; BN intrinsic phonon modes supply additional absorption/emission centers; and C–O/O–H characteristic vibrations together with TOCNF’s micro-nano architecture expand the effective radiative surface through multiple scattering pathways. BN addition also smooths the emissivity spectrum by filling absorption valleys, averting radiation-efficiency loss from atmospheric attenuation (Figure 4d) [6,41].

### 3.5. Thermal Performances of the Coatings

**Figure 5:**
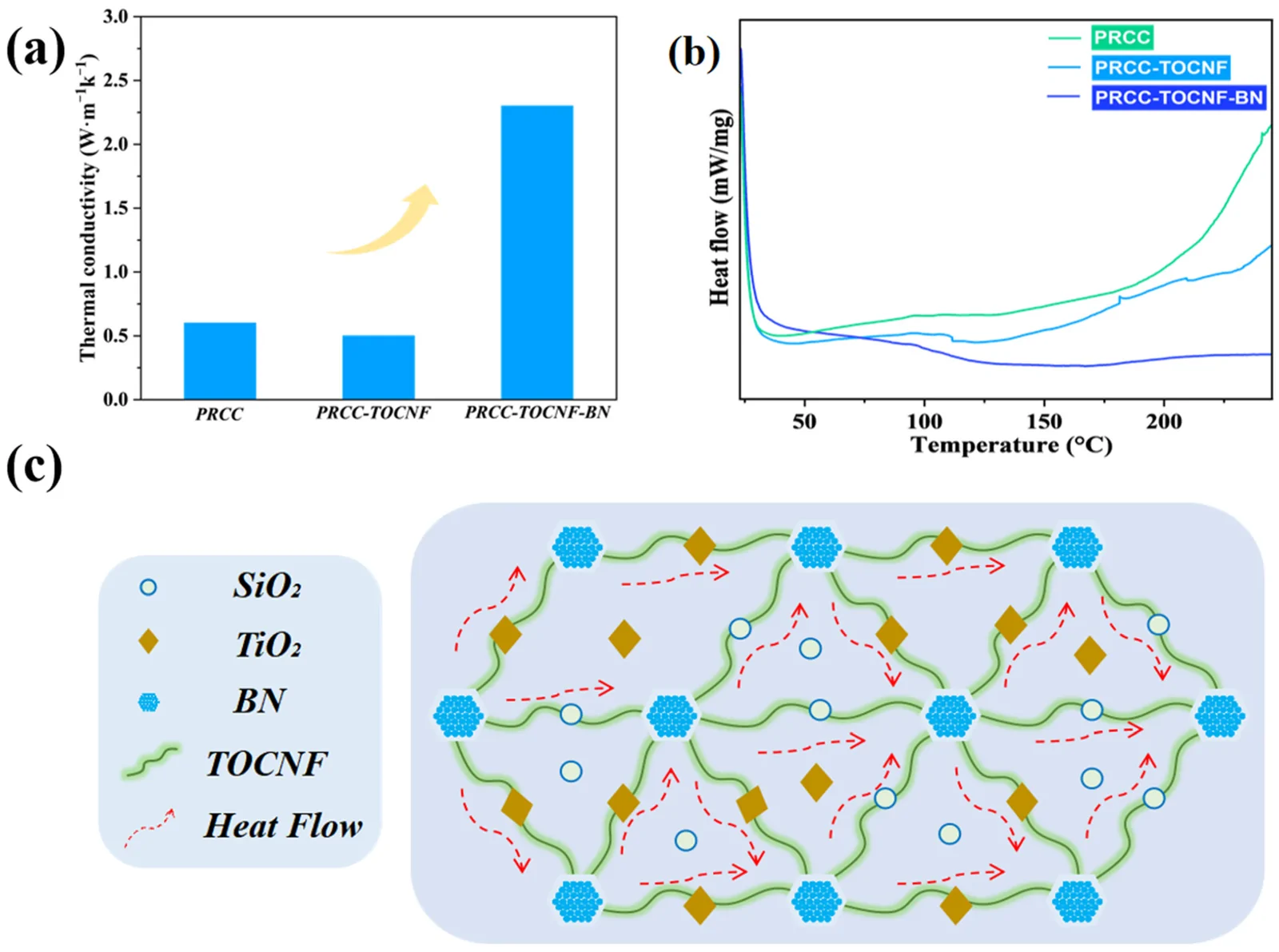
Thermal performances of the coatings: (**a**) thermal conductivity; (**b**) DSC curves; (**c**) schematic illustration of the thermal conductive network.

Figure 5 details the thermal conduction behavior and underlying heat-transfer mechanisms of the prepared coatings. As shown in Figure 5a, neat PRCC exhibits a modest thermal conductivity of 0.58 ± 0.03 W·m^−1^·K^−1^, reflecting the intrinsically poor heat-transport capacity of the pure acrylic matrix. Adding TOCNF alone (PRCC-TOCNF) slightly lowers the value to 0.48 ± 0.04 W·m^−1^·K^−1^, a consequence of TOCNF’s own low thermal conductivity of 0.05–0.10 W·m^−1^·K^−1^ and the extra interfacial thermal resistance generated by extensive hydrogen bonding between TOCNF and the polymer [29]. In stark contrast, PRCC-TOCNF-BN delivers a thermal conductivity of 2.28 ± 0.15 W·m^−1^·K^−1^—293% and 375% above the values for neat PRCC and PRCC-TOCNF, respectively. At only 5 wt% BN loading, the coating reaches a competitive thermal conductivity via a straightforward waterborne Pickering emulsion route. By comparison, traditional ceramic-filled radiative cooling materials generally demand 40–60 wt% filler to approach similar thermal conductivity, incurring higher material cost and degraded coating processability [18]. DSC curves (Figure 5b) reflect the thermal stability differences among the coatings. Through the synergistic contribution of TOCNF and BN, PRCC-TOCNF-BN exhibits a discernible upward shift in thermal decomposition temperature relative to both control samples, confirming improved thermal durability for practical deployment.

The heat conduction mechanism is illustrated schematically in Figure 5c. Leveraging the Pickering emulsion template, amphiphilic TOCNF fulfills dual roles—emulsifier and structural scaffold—effectively curbing BN nanosheet agglomeration and promoting their partial alignment during shear emulsification and film formation. Because the platelets are anchored on three-dimensionally distributed droplet interfaces, the resulting BN network is quasi-3D and interconnected: partially in-plane-biased sheets are bridged by extensive edge-to-face contacts, which simultaneously provide efficient in-plane heat spreading and continuous through-plane phonon pathways [17].

**Figure 6:**
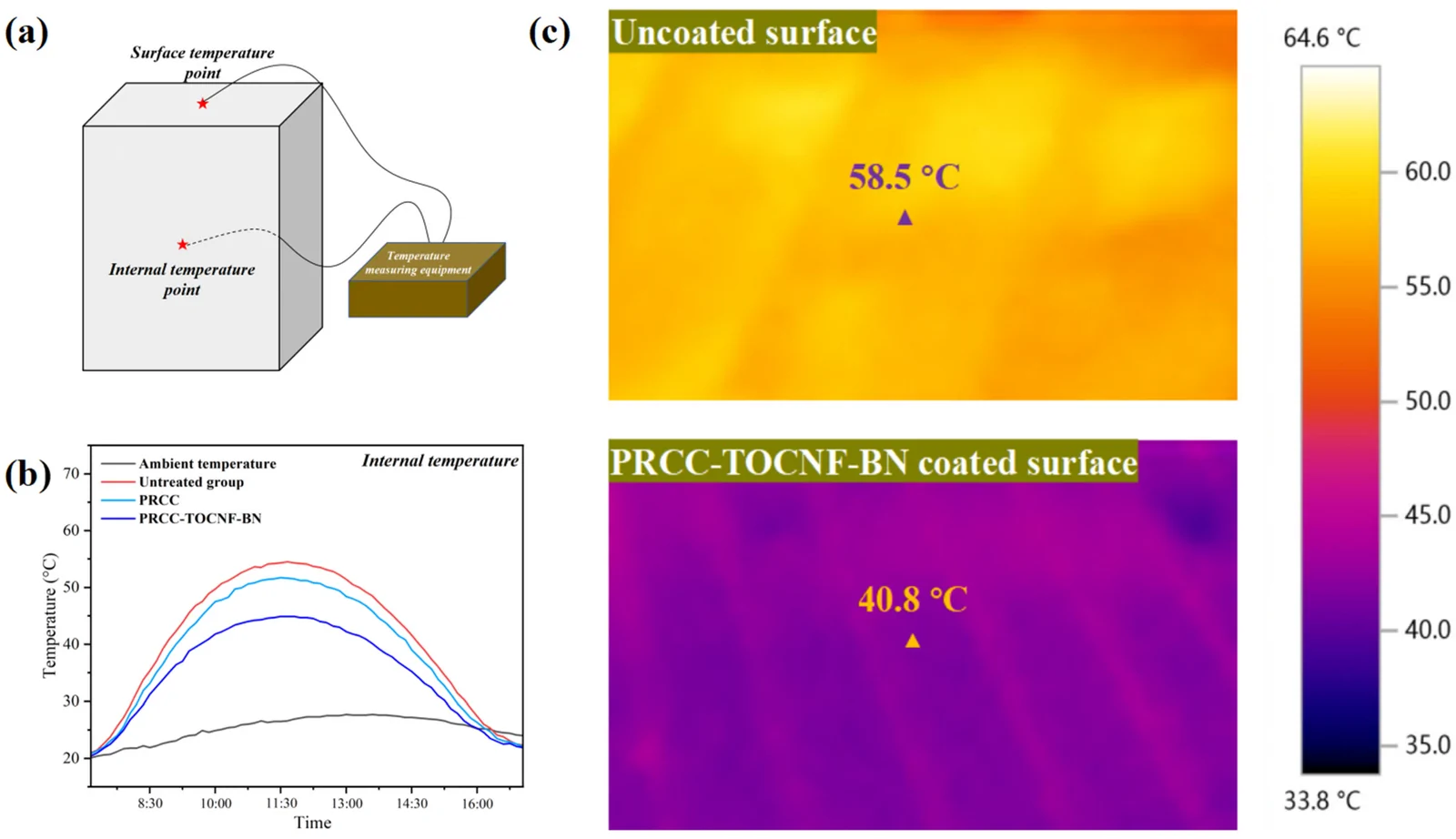
Cooling performances of the coatings: (**a**) model diagram for outdoor temperature testing; (**b**) internal temperature of the model versus time; (**c**) infrared thermal images.

Outdoor cooling performance was assessed with model cabinet tests under natural sunlight in Suzhou, China; the experimental arrangement and findings are summarized in Figure 6. Figure 6a depicts the outdoor test platform. Three identical metal cabinets were employed: one fully coated with PRCC-TOCNF-BN, one coated with pristine PRCC, and one left bare. All stood on polyurethane foam insulation to suppress ground conduction; testing proceeded under clear skies with peak irradiance above 850 W/m^2^ and a maximum ambient temperature of 27.7 °C.

Temporal evolution of internal cabinet temperatures is plotted in Figure 6b. Throughout the 8:30–16:00 recording window, the bare cabinet consistently recorded the highest temperature, peaking at 54.6 °C. The PRCC-coated cabinet offered moderate relief, with its peak falling to 51.7 °C. Most strikingly, the PRCC-TOCNF-BN cabinet achieved the lowest peak internal temperature of 45.0 °C–9.6 °C below the bare reference and 6.7 °C below the PRCC-only unit.

Infrared thermographic images (Figure 6c) visually corroborate the surface-temperature differences. The bare cabinet surface reached 58.5 °C under solar irradiation, whereas the PRCC-TOCNF-BN coated surface dropped to 40.8 °C, a reduction of 17.7 °C. This pronounced cooling stems from the coating’s combined attributes: high solar reflectance (~87%) blocks most incoming solar heat, high ATSW emissivity (~93%) continuously radiates internal heat toward space, and the continuous BN thermal network rapidly exports accumulated heat through the coating thickness [5]. The outdoor data validate that the TOCNF-mediated Pickering emulsion strategy endows the radiative cooling coating with improved cooling performance under a purely solar-driven thermal load [11].

## 4. Conclusions

In summary, we have established a nanocellulose-mediated Pickering emulsion route for fabricating radiative cooling coatings in which thermal conductivity and optical performance are simultaneously enhanced. By deploying TEMPO-oxidized cellulose nanofibrils (TOCNF) as interfacial stabilizers, hexagonal boron nitride (BN) nanosheets were homogeneously distributed within a waterborne acrylic matrix, producing a continuous, three-dimensionally interconnected thermally conductive network. The optimized PRCC-TOCNF-BN nanocomposite registers a thermal conductivity of 2.28 ± 0.15 W·m^−1^·K^−1^, a 293% improvement over the neat polymer reference (0.58 W·m^−1^·K^−1^). This performance gain traces directly to the Pickering templating mechanism, which maximizes interfacial contact between BN nanosheets while suppressing agglomeration-driven phonon scattering. The thermal conductivity enhancement is attained without any compromise to radiative cooling optical function: the ternary composite retains high solar reflectance (~87% across 0.3–2.5 μm) and strong ATSW emissivity (~93% across 8–13 μm). The synergy of Mie-scattering TiO_2_, phonon-polaritonic SiO_2_, and BN, complemented by the hierarchical micro-nano architecture generated by TOCNF, underpins the broadband optical performance essential for sub-ambient cooling. XRD and SEM results confirms that TOCNF fulfills two distinct functions: first, as a Pickering emulsifier that forestalls filler agglomeration and secures uniform spatial distribution; second, as a transient scaffold that induces partial alignment and 3D interconnection of BN nanosheets during shear-driven film formation. This work resolves a pivotal limitation in polymer-based radiative cooling materials—the inherent trade-off between thermal conductivity and optical efficacy—and establishes a scalable, waterborne processing avenue for multifunctional coatings targeting high-power electronics, concentrated photovoltaics, and industrial thermal management.

## Data Availability

The original contributions presented in this study are included in the article/Supplementary Material. Further inquiries can be directed to the corresponding author.

## Supplementary Materials

The following supporting information can be downloaded at: https://www.mdpi.com/article/10.3390/polym18202591/s1. Figure S1: Thermal conductivities of the PRCC-TOCNF-BN coatings with BN loadings of 0, 1, 3, 5, and 7.5 wt%; Table S1: Raw thermal-diffusivity, density and specific-heat data of the coatings at 25 °C. Values are mean ± standard deviation over three independently prepared specimens.

## References

[1] Jia F., Yin X., Zhang Y., Fang J., Zong S., Cao F., Chen B., Wang X. (2026). Time-dependent range degradation in low-carbon electric vehicle thermal management systems: A quantitative assessment across China. Renew. Energy.

[2] Arabi M., Ostovan A., Li J., Wang X., Zhang Z., Choo J., Chen L. (2021). Molecular Imprinting: Green Perspectives and Strategies. Adv. Mater..

[3] Bai L., Liu L., Esquivel M., Tardy B.L., Huan S., Niu X., Liu S., Yang G., Fan Y., Rojas O.J. (2022). Nanochitin: Chemistry, Structure, Assembly, and Applications. Chem. Rev..

[4] Boretti A. (2025). Advanced Battery Thermal Management: A Review of Materials, Cooling Systems, and Intelligent Control for Safety and Performance. Energy Storage.

[5] Zhao B., Hu M., Ao X., Chen N., Pei G. (2019). Radiative cooling: A review of fundamentals, materials, applications, and prospects. Appl. Energy.

[6] Raman A.P., Anoma M.A., Zhu L., Rephaeli E., Fan S. (2014). Passive radiative cooling below ambient air temperature under direct sunlight. Nature.

[7] Ma Z., Feng Y., Song A.Y., Dinh T., Hong M., Bell J., Hou B., Song P., Zheng W. (2025). A Scalable, Durable, Fire-Safe All-Day Passive Radiative Cooling Coating for Sustainable Buildings. Adv. Funct. Mater..

[8] Hu D., Sun S., Du P., Lu X., Zhang H., Zhang Z. (2022). Hollow Core-Shell Particle-Containing Coating for Passive Daytime Radiative Cooling. Compos. Part A Appl. Sci. Manuf..

[9] Xin Y., Li C., Gao W., Chen Y. (2025). Emerging colored and transparent radiative cooling: Fundamentals, progress, and challenges. Mater. Today.

[10] Xie A.-Q., Qiu H., Jiang W., Wang Y., Niu S., Zhang K.-Q., Ho G.W., Wang X.-Q. (2025). Recent advances in spectrally selective daytime radiative cooling materials. Nano-Micro Lett..

[11] Wang H., Xie B., Li C. (2025). Review on operation control of cold thermal energy storage in cooling systems. Energy Built Environ..

[12] Huang M.-C., Yang M., Guo X.-J., Xue C.-H., Wang H.-D., Ma C.-Q., Bai Z., Zhou X., Wang Z., Liu B.-Y. (2023). Scalable multifunctional radiative cooling materials. Prog. Mater. Sci..

[13] Huang S., You T., Huang Z. (2025). Multi-physics analyses on a passive radiative cooling coating for performance improvement of high-speed railways in hot regions. Renew. Energy.

[14] Zhang H., Fan D. (2020). Improving Heat Dissipation and Temperature Uniformity in Radiative Cooling Coating. Energy Technol..

[15] Zhan Z., Jing Y., Peng Z., Liu Q., Xie X. (2025). Applicability of air-cooled data center cooling systems in different locations across China: A simulation-based investigation. Energy.

[16] Han L., Yang F., Meng Q., Yang X., Li J. (2025). Passive radiative cooling ZrO_2_/graphene composite coating with high emissivity and enhanced thermal conductivity for heat dissipation. Ceram. Int..

[17] He W., Shang H., Cao Y., Yu Y. (2025). Development and application of superhydrophobic coatings with high thermal conductivity and self-healing properties: Synergistically enhancing condensation heat transfer through surface modification. Compos. Part A Appl. Sci. Manuf..

[18] Wu M.-Z., Zhou Z.-H., Hao T.-T., Liu D.-P., Duan L.-L., Qi J.-W., Xie H.-L. (2025). Conductive Liquid Crystal Elastomers Enabled by Deep Eutectic Solvent for Temperature Warning Systems. ACS Appl. Mater. Interfaces.

[19] Ji R., Wang S., Zhao X., Zhang J., Zou Y., Chen G., Wang Y., Huang Y., Ouyang J., Jia D. (2024). TiO_2_-BN/CNTs coating with radiative cooling and reduced friction. Chem. Eng. J..

[20] Umapathi R., Rethinasabapathy M., Kakani V., Kim H., Park Y., Kim H.K., Rani G.M., Kim H., Huh Y.S. (2025). Hexagonal boron nitride composite film based triboelectric nanogenerator for energy harvesting and machine learning assisted handwriting recognition. Nano Energy.

[21] Lu X., Lin Y., Chen W., He H., Zhang B., Yu Y. (2025). Facile fabrication of dielectric carbon fiber@hexagonal boron nitride nanosheets core–shell hierarchical structure towards highly efficient microwave absorption. Appl. Surf. Sci..

[22] Lai X., Li G., Coe A.M., Pixley J.H., Watanabe K., Taniguchi T., Andrei E.Y. (2025). Moiré periodic and quasiperiodic crystals in heterostructures of twisted bilayer graphene on hexagonal boron nitride. Nat. Mater..

[23] Lee J., Yoon J., Jeon M., Oh S.-G. (2025). Aramid-based composite nanofiber separator coated with thermal conductive layer containing hexagonal boron nitride for stable lithium metal batteries from dendritic Li growth. Chem. Eng. J..

[24] Wu J., Ma G.H. (2016). Recent Studies of Pickering Emulsions: Particles Make the Difference. Small.

[25] Li C., Ma S., Han X., Sun Y., Li C., Zheng K., Xin Y., Li R. (2025). Malleable, Self-Healing, and Highly Thermally Conductive Interface Material Enabled by Interfacial Networks for Thermal Management. ACS Appl. Mater. Interfaces.

[26] Zhang S., Guo H., Zhang B., Ding K., Li H., Ai S., Shan Y., Ding S. (2025). A composite coating film based on the interfacial interlocking symbiosis of nanolignin Pickering emulsion and pectin matrix for perishable fruit preservation. Chem. Eng. J..

[27] Lin Z., Miao L., Song H., Wang S., Zhen J., Lv W. (2025). Rod-like boron nitride based-coating for subambient and above-ambient passive radiative cooling. Appl. Surf. Sci..

[28] Zheng Y., Oguzlu H., Baldelli A., Zhu Y., Song M., Pratap-Singh A., Jiang F. (2022). Sprayable cellulose nanofibrils stabilized phase change material Pickering emulsion for spray coating application. Carbohydr. Polym..

[29] Nakamitsu K., Yokota S. (2026). Controlled surface modification and medium-guided assembly of bacterial cellulose-based nanofibrils for the preparation of water-repellent thin films. Int. J. Biol. Macromol..

[30] Krajnc M., Alič B., Malnarič I., Šebenik U. (2025). A mathematical model describing elastic response of hybrid hydrogel from TEMPO-oxidized cellulose nanofibrils and graphene oxide. Chem. Eng. J..

[31] Liu Y., Wen W., Shen C., Qiu X., Liu Y., Qing X., Chen Z., Gao Y., Zhang H., Zhang Z. (2025). A Flexible, Wear-Resistant, Ultrablack Coating Modified by an Antireflective Layer Prepared with a Dry–Wet Hybrid Deposition Methodology. ACS Appl. Mater. Interfaces.

[32] (2022). Standard Test Method for Thermal Diffusivity by the Flash Method.

[33] Salajková M., Berglund L.A., Zhou Q. (2012). Hydrophobic cellulose nanocrystals modified with quaternary ammonium salts. J. Mater. Chem..

[34] Park Y.G., Lee Y.H., Rahman M.M., Park C.C., Kim H.D. (2015). Preparation and properties of waterborne polyurethane/self-cross-linkable fluorinated acrylic copolymer hybrid emulsions using a solvent/emulsifier-free method. Colloid Polym. Sci..

[35] Luo J., Song T., Han T., Qi H., Liu Q., Rosenau T. (2024). Multifunctional roles of TEMPO-oxidized cellulose nanofibrils on the enhancement of mechanical and conductive properties of acrylic-based hydrogels for temperature response and human motion sensing. Chem. Eng. J..

[36] Gou J., Liu W., Tang A., Xie H. (2021). A phosphorylated nanocellulose/hydroxypropyl methylcellulose composite matrix: A biodegradable, flame-retardant and self-standing gel polymer electrolyte towards eco-friendly and high safety lithium ion batteries. Eur. Polym. J..

[37] Luo K., Wang H., Li E., Tang B., Yuan Y. (2026). Interface engineering to construct BN@SiO_2_ multilayer structure for thermal interface materials with high thermal conductivity and low dielectric loss. Appl. Surf. Sci..

[38] Wang H., Qin X., Feng Y., Huang B., Zou X., Quan J., Lan P., Lan L. (2025). Preparation of high-performance waterborne acrylic-based adhesive for soft porcelain veneer molding. Mater. Today Commun..

[39] Diercks C.S., Yaghi O.M. (2017). The atom, the molecule, and the covalent organic framework. Science.

[40] Gong Q., Chun Wong H., Chen J., Li P., Lu L. (2024). Solar-driven adaptive radiative cooling coating with polymer carbon dots-enhanced photoluminescence for urban skin. Chem. Eng. J..

[41] Zhang G., Pang H., Zhang Z., Wang F., Sun X., Yang Z., Song J., Xu Z., Li X., Dong Y. (2025). Scalable industrial production line establishment and performance investigation of strong adhesion commercial radiative cooling coating. Energy Build..

[42] Chen G., Wang Y., Qiu J., Cao J., Zou Y., Wang S., Jia D., Zhou Y. (2021). A facile bioinspired strategy for accelerating water collection enabled by passive radiative cooling and wettability engineering. Mater. Des..

