# Supplementary material for: Construction of Uniform Thermal Network in Passive Radiative Cooling Coatings by Nanocellulose-Mediated Pickering Emulsion Template
Source: Polymers (Basel). 2026 Oct 21;18(20):2591. doi: 10.3390/polym18202591 (PMC13709207; doi:10.3390/polym18202591)
Supplement: Supplementary file 1 [file polymers-18-02591-s001.zip › polymers-4536507-supplementary.pdf]

Construction of Uniform Thermal Network in Passive Radiative Cooling Coatings by Nanocellulose-Mediated Pickering Emulsion Template

Huiwen Pang [1,2,3,*], Jiaqi Zhu [2], Zenghui Xu [1], Tong Wang [3], Xiang Li [1], Xing Chen [3] and Niancheng Hong [1]

Thermal conductivities of the PRCC-TOCNF-BN coatings

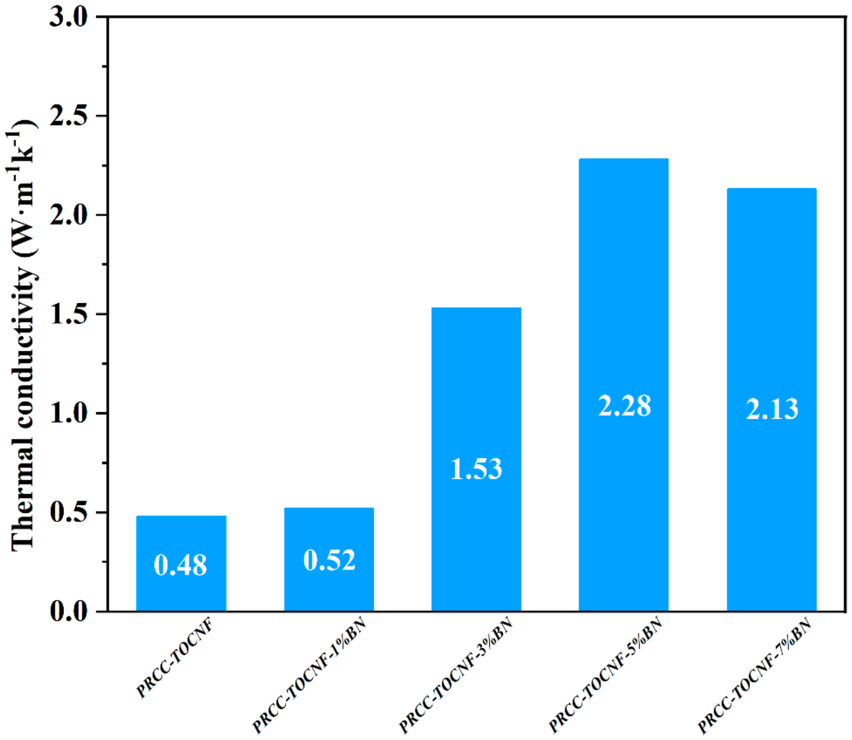

Figure S1. Thermal conductivities of the PRCC-TOCNF-BN coatings with BN loadings of 0, 1, 3, 5, and 7.5 wt%.

Raw data of the coatings

Table S1. Raw thermal-diffusivity, density and specific-heat data of the coatings at 25 °C. Values are mean ± standard deviation over three independently prepared specimens

**Specimen**	**α (mm²·s⁻¹)**	**ρ (g·cm⁻³)**	**cp (J·g⁻¹·K⁻¹)**	**λ = α·ρ·cp (W·m⁻¹·K⁻¹)**
PRCC	0.331 ± 0.010	1.39 ± 0.02	1.27 ± 0.04	0.58 ± 0.03
PRCC-TOCNF	0.273 ± 0.008	1.39 ± 0.02	1.27 ± 0.04	0.48 ± 0.04
PRCC-TOCNF-BN	1.294 ± 0.039	1.43 ± 0.02	1.23 ± 0.04	2.28 ± 0.15

Optical Performance Test

The average solar reflectance ($\overline{R}_{solar}$)and ATSW emittance ($\overline{\varepsilon}_{ATSW}$) can be calculated as following:

$$\overline{R}_{solar} = \frac{\int_{0.3}^{2.5} I_{AM1.5}(\lambda) R(\lambda) d\lambda}{\int_{0.3}^{2.5} I_{AM1.5}(\lambda) d\lambda}$$

And

$$\overline{\varepsilon}_{ATSW} = \frac{\int_{8}^{13} I_{BB}(\lambda,T) \varepsilon(\lambda) d\lambda}{\int_{8}^{13} I_{BB}(\lambda,T) d\lambda}$$

where λ represents the wavelength, $I_{\mathrm{AM1.5}}$ represents the AM 1.5 global solar intensity, $R(\lambda)$ and $\varepsilon(\lambda)$ represent the reflectance and emittance at the wavelength λ. I_{BB} represents the monochromatic emittance of the radiative, and the calculation of I_{BB} is as follows:

$$I_{BB}(\lambda,T) = \frac{2hc^2}{\lambda^5} \frac{1}{\exp(\frac{hc}{\lambda k_{\mathrm{B}} T}) - 1}$$

According to Kirchhoff's law of radiation, the absorptivity of the radiative cooling coating can be equated to its emissivity, denoted as $\varepsilon(\lambda)=A(\lambda)=1-R(\lambda)-T(\lambda)$. For opaque radiative cooling coatings, the transmittance $T(\lambda)$ is equal to zero. Therefore, the emittance $\varepsilon(\lambda)=A(\lambda)=1-R(\lambda)$.
